# Exploring Small Molecules Targeting Protein–Protein Interactions (PPIs): Advancements and Future Prospects

**DOI:** 10.3390/ph16121644

**Published:** 2023-11-23

**Authors:** Sachin P. Patil

**Affiliations:** NanoBio Lab, School of Engineering, Widener University, Chester, PA 19013, USA; spatil@widener.edu; Tel.: +1-610-499-4492

## 1. Introduction

This Special Issue of *Pharmaceuticals* is dedicated to the clinically relevant, intricate realm of “Small Molecules Targeting Protein–Protein Interactions (PPIs): Current Strategies for the Development of New Drugs”. Within the dynamic landscape of drug discovery, the quest for innovative therapeutic avenues continues to drive the scientific community, and our present Special Issue delves into one such important therapeutic domain that holds immense promise—PPIs. Specific focus on small molecules as modulators of PPIs will make new inroads in drug development, offering unprecedented opportunities to reshape the pharmacological landscape.

PPIs play a pivotal role in cellular functions and the regulation of various biological processes [1]. The dysregulation of these protein interactions is often associated with a number of diseases; thus, harnessing these PPIs for drug discovery purposes represents an unprecedented therapeutic opportunity. Indeed, PPIs represent a vast source of novel therapeutic targets, with the size of the human interactome reaching an estimated number of over 300,000 interactions and counting [2]. This is welcome news considering the disappointingly well-known decrease in drug discovery and development efficiency in recent years [3], primarily due to the exhaustion of effective targets for traditional drug design that focuses on single proteins as opposed to PPIs.

Traditionally, *Interactome Engineering* through PPI modulation falls within the realm of primarily large biotherapeutics including monoclonal antibodies, protein fragments, and macrocyclic peptides. The small-molecule tractability of PPIs, however, is on the rise, with the first such small-molecule drugs entering clinical trials recently [4], providing the much-needed impetus for this traditionally challenging area of research. Therefore, the focus of this Special Issue is on this burgeoning field of small molecules as modulators of PPIs—a topic that holds immense promise for the future of drug design.

Indeed, the PPI field has witnessed a paradigm shift in recent years towards the development of small-molecule inhibitors as well as stabilizers [5], owing to their distinct advantages over larger protein-based counterparts. Specifically, the small molecules offer improved bioavailability, reduced immunogenicity, and enhanced cell permeability, thereby addressing some of the challenges associated with targeting PPIs with large-protein therapeutics [6]. In this context, the articles featured in this Special Issue delve into the current strategies employed in the discovery and development of such small molecules targeting PPIs, providing valuable insights into the innovative approaches driving this field forward.

## 2. Highlighted Papers from the Special Issue

- In this Special Issue, we witness a confluence of cutting-edge research and insightful review articles, providing a inclusive overview of the current state of small-molecule PPI modulators.
- In “*Dehydroeburicoic Acid, a Dual Inhibitor against Oxidative Stress in Alcoholic Liver Disease*” by Shasha Cheng et al. (Contribution 1), the study explores the potential of dehydroeburicoic acid (DEA) as a dual inhibitor against oxidative stress in alcoholic liver disease. The work unveils DEA’s role in disrupting the Keap1–Nrf2 protein–protein interaction and inhibiting GSK3β, showcasing its promise in mitigating alcoholic liver disease by restoring Nrf2 activity and promoting antioxidant genes.
- In “Rational Strategy for Designing Peptidomimetic Small Molecules Based on Cyclic Peptides Targeting Protein–Protein Interaction between CTLA-4 and B7-1” by Kumiko Tsuihiji et al. [Contribution 2], a rational two-step strategy for designing small-molecule compounds targeting the protein–protein interaction between CTLA-4 and B7-1 is presented. Leveraging inhibitory cyclic peptides to inform the design of small molecules, the study successfully generates compounds with good IC50 values against CTLA-4.
- In “Broad-Spectrum Small-Molecule Inhibitors of the SARS-CoV-2 Spike—ACE2 Protein–Protein Interaction from a Chemical Space of Privileged Protein Binders” by Sung-Ting Chuang and Peter Buchwald [Contribution 3], a library of small-molecule inhibitors targeting the SARS-CoV-2 spike—ACE2 PPI is explored. The study identifies promising inhibitors, including organic dyes and novel drug-like compounds, with potential broad-spectrum activity against COVID-19 variants of concern.
- In “*Rational Design by Structural Biology of Industrializable, Long-Acting Antihyperglycemic GLP-1 Receptor Agonists*” by Lei Sun et al. [Contribution 4], the challenge of improving the stability of GLP-1 receptor agonists for treating type II diabetes is tackled. Their rational design, informed by structural biology, leads to the development of GLP-1 receptor agonists with extended hypoglycemic effects lasting over 24 h, providing crucial insights into drug design in the field of diabetes therapeutics.
- In “*Machine-Learning Guided Discovery of Bioactive Inhibitors of PD1-PDL1 Interaction*” by Sachin P. Patil et al. [Contribution 5], a machine learning approach is employed to discover bioactive inhibitors of PD1–PDL1 interaction, a crucial target in cancer immunotherapy. The study combines ML models with molecular docking to identify and experimentally validate two active molecules, CRT5 and P053, with promising potential as PD1–PDL1 inhibitors.
- In “*Synthetic Design and Biological Evaluation of New p53-MDM2 Interaction Inhibitors Based on Imidazoline Core*” by Daniil R. Bazanov et al. [Contribution 6], a two-step synthesis approach for imidazoline-based alkoxyaryl compounds that efficiently inhibit p53-MDM2 PPI is described. These compounds demonstrate significant upregulation of p53 and p53-inducible proteins in various cancer cell lines, showcasing their potential as anti-cancer agents.
- In “*Targeting Protein–Protein Interactions to Inhibit Cyclin-Dependent Kinases*” by Mark Klein [Contribution 7], the comprehensive review explores the landscape of targeting protein–protein interactions involving cyclin-dependent kinases (CDKs). Focusing on CDKs 2, 4, 5, and 9, the review highlights promising lead molecules in the pursuit of selective CDK inhibitors, laying the foundation for further discoveries in this critical area of cancer therapeutic strategies.

## 3. Conclusions 

In conclusion, this Special Issue highlights successes, challenges, and emerging opportunities in the PPI field, with particular emphasis on the various strategies, both experimental and computational, being utilized for the development of small-molecule modulators of clinically relevant PPIs. It serves as a testament to the evolving landscape of drug discovery and the pivotal role small molecules play in modulating PPIs. We invite our readers to embark on this intellectual journey, exploring the frontiers of small molecules targeting PPIs and envisioning a future where these molecular architects shape the next generation of effective therapeutic interventions against a variety of human disorders.
